# Conservation and Loss of a Putative Iron Utilization Gene Cluster among Genotypes of *Aspergillus flavus*

**DOI:** 10.3390/microorganisms9010137

**Published:** 2021-01-09

**Authors:** Bishwo N. Adhikari, Kenneth A. Callicott, Peter J. Cotty

**Affiliations:** 1 Chemical/Biological Technologies Department, Research and Development Directorate, Defense Threat Reduction Agency, Fort Belvoir, VA 22060, USA; 2 United States Department of Agriculture, Agriculture Research Service, 416 W Congress St, First Floor, Tucson, AZ 85701, USA; 3 College of Food Science and Engineering, Ocean University of China, Qingdao 266003, Shandong, China

**Keywords:** iron, gene cluster, deletion, *Aspergillus flavus*, iron utilization gene cluster, evolution

## Abstract

Iron is an essential component for growth and development. Despite relative abundance in the environment, bioavailability of iron is limited due to oxidation by atmospheric oxygen into insoluble ferric iron. Filamentous fungi have developed diverse pathways to uptake and use iron. In the current study, a putative iron utilization gene cluster (IUC) in *Aspergillus flavus* was identified and characterized. Gene analyses indicate *A. flavus* may use reductive as well as siderophore-mediated iron uptake and utilization pathways. The ferroxidation and iron permeation process, in which iron transport depends on the coupling of these two activities, mediates the reductive pathway. The IUC identified in this work includes six genes and is located in a highly polymorphic region of the genome. Diversity among *A. flavus* genotypes is manifested in the structure of the IUC, which ranged from complete deletion to a region disabled by multiple indels. Molecular profiling of *A. flavus* populations suggests lineage-specific loss of IUC. The observed variation among A. flavus genotypes in iron utilization and the lineage-specific loss of the iron utilization genes in several *A. flavus* clonal lineages provide insight on evolution of iron acquisition and utilization within *Aspergillus* section *Flavi*. The potential divergence in capacity to acquire iron should be taken into account when selecting *A. flavus* active ingredients for biocontrol in niches where climate change may alter iron availability.

## 1. Introduction

Iron is an essential element for the majority of organisms, where it serves as cofactor for enzymatic reactions and catalyst for electron transport systems. Despite relative abundance in most environments, bioavailability of iron is limited due to oxidation by atmospheric oxygen into insoluble ferric (Fe^3+^) oxyhydroxides, and iron-dependent organisms must reduce ferric iron to its ferrous (Fe^2+^) state prior to absorbtion. At the same time, concentrations of this metal must be tightly regulated since it can catalyze the formation of highly reactive oxygen species and cause tissue damage [1,2,3]. In most organisms, cellular iron homeostasis is designed to tightly regulate the iron supply to prevent excess accumulation. Because of the twin needs of iron uptake and iron regulation, iron-dependent organisms have evolved tightly regulated acquisition and storage strategies [4,5], and iron often serves as a signal for gene expression involved in iron uptake and storage. Many prokaryotic and eukaryotic pathogens require iron for virulence [6,7] and employ multiple pathways for iron uptake, utilization, and storage [8]. 

Under iron starvation, many microorganisms, including fungi, utilize low-molecular mass (<1000 Da) compounds with high iron affinity termed ‘siderophores’ [9,10] for iron acquisition and storage. Biosynthesis of ferric-specific siderophores is regulated by iron availability [11]. High iron concentrations repress siderophore production. Most species of *Aspergillus* produce several hydroxamate-type siderophores, which sequester iron in the surrounding environment [12]. In addition to iron acquisition, siderophores play important roles in intracellular iron storage [13] and fungal virulence [14]. Studies of many fungal genome sequences have shown that genes for siderophore-iron transporters are widely conserved in the fungal kingdom, even in taxa that do not secrete siderophores such as *Sacharomyces cerevisiae*, *Candida* spp., and *Cryptococcus neoformans* [11,15,16,17,18].

Understanding of iron uptake and utilization by plant and animal pathogens and the underlying molecular mechanisms has recently improved [8,11,19,20,21,22]. Iron uptake mechanisms in the Basidiomycota (*Ustilago maydis*) and Ascomycota (*S. pombe*, *Neurospora crassa, Aspergillus nidulans*, *A. fumigatus*, *S. cerevisiae*, and *Candida albicans*) have been well described [8,22,23]. *Saccharomyces cerevisiae* has two different high-affinity iron uptake systems [8]. The first is the reductive iron assimilation (RIA) pathway, in which ferric iron is reduced to ferrous iron by cell surface reductase activity and transported across the plasma membrane by a high-affinity iron permease (Ftr1)–multicopper ferroxidase (Fet3) complex [24,25]. The second imports siderophores-bound iron via cell surface transporters [26]. Presence of both siderophore-mediated iron uptake and RIA has been functionally validated in *A. fumigatus* [11]. Though the presence of membrane-bound reductive iron assimilatory systems has been reported in a broad array of fungi [8,21,27,28,29,30,31,32], the structure and diversity of iron uptake and utilization systems in *A. flavus* remain unknown.

Members of the genus *Aspergillus* are ubiquitous in nature, frequently occurring as saprotrophs on decaying organic matter. *Aspergillus* section *Flavi* comprises important opportunistic pathogens of both plants and animals. *Aspergillus fumigatus* and *Aspergillus niger*, common causes of aspergillosis, contain well studied iron uptake and metabolism systems [18,22,33,34,35]. The iron metabolism system of *A. nidulans*, a rare cause of aspergillosis in humans, is also described in detail [22,36,37,38]. In contrast to several fungal pathogens [39,40], both *A. fumigatus* and *A. nidulans* lack the ability to uptake iron from host sources like heme, ferritin, and transferrin. Instead, both *A. fumigatus* and *A. nidulans* use two high-affinity iron uptake systems, siderophore-assisted iron uptake and reductive iron assimilation (RIA), which are induced upon iron starvation [18,36]. The molecular mechanisms underlying iron uptake in *A. flavus,* another common cause of human aspergillosis, and variation among *A. flavus* isolates in iron utilization are not well studied.

Certain genotypes of *A. flavus* produce aflatoxins that contaminate food crops, including maize, peanuts, and tree nuts [41,42]. Although incidences and severities of crop contamination are influenced by the genetic structure of *A. flavus* populations [43], environmental factors and host nutrient content also play important roles in the pathogenicity of *A. flavus* to plants [44,45,46]. *Aspergillus flavus* is common in soil environments where iron availability is limited. Iron also has a strong influence on growth [47] and expression of genes involved in aflatoxin biosynthesis [48]. Metal ions play an important role in eukaryote transcription [49], and iron influences cellular processes through increased production of RNA and induction of gene expression [50,51]. 

The current study identified and characterized variation in groups of genes of potential use in iron utilization in *A. flavus*. These genes are grouped together forming a putative cluster that is referred to here as Iron Utilization Cluster (IUC). Analyses of the genes suggest *A. flavus* may have two iron uptake and utilization pathways: the reductive and the siderophore-mediated pathways. Lineage-specific loss of iron utilization genes in several *A. flavus* clonal lineages provides insight on evolution of this genomic region within *Aspergillus* section *Flavi*. The ferroxidation and iron permeation pathway, in which iron transport depends on the coupling of the two activities, is here described for *A. flavus*.

## 2. Materials and Methods

### 2.1. Fungal Isolates and Culture Conditions

Five *A. flavus* isolates with and without ability to produce aflatoxins were used in the initial stages of the study (Table 1). Isolates were characterized and described previously [52,53]. Isolates belonging to different vegetative compatibility groups (VCGs) were selected to ensure genotypic diversity. Wildtype isolates from silica gel storage were cultivated on 5/2 agar (5% V8 juice, 2% agar, pH 5.2). Wildtype isolates were first transferred by single spore and then maintained in 4-mL vials by suspending plugs of 5/2 agar with abundant sporulation in sterile distilled water. Aspergillic acid production was assessed on *Aspergillus flavus* and *parasiticus* agar (AFPA) medium as previously described [54]. AFPA is used to identify fungi in *Aspergillus* section *Flavi*. [54]. When grown on AFPA agar (10g Bacto agar, 20g yeast extract, 10 g Bacto peptone and 0.5 g ferric ammonium citrate in 500 mL of water), *A. flavus* and *A. parasiticus* develop characteristic orange color on the reverse of plates from the reaction of ferric iron from ferric ammonium citrate (FAC) with aspergillic acid molecules [54]. Response of *A. flavus* isolates to different iron concentrations was tested on chemically defined Czapek’s agar medium with 4, 2, 0.5, and 0 mM concentration of FAC. The medium was autoclaved (121 °C; 20 min), and filter-sterilized FAC solution was added prior to pouring. Reverse sides of plates were photographed after 5 days for color assessment. The color intensity was measured using ImageJ software [55]. At least three pictures were taken for each strain and FAC concentration. 

### 2.2. Gene Identification and Characterization 

IUCs were identified while analyzing the deleted regions from *A. flavus* through comparisons with *A. oryzae* RIB40 [56]. Deletions were predicted using DELLY [57], a variant detection program that predicts deletions by mapping paired-end reads from an interrogated isolate to a reference genome. Regions polymorphic in the five *A. flavus* isolates were further annotated using MAKER [58]. Deleted regions were characterized in reference to corresponding regions from the annotated genome of *A. oryzae* RIB40. Expression of genes in IUC was measured by mapping the transcripts from *A. flavus* NRRL3357 [59] to genomic regions from different *A. flavus* genotypes. Sequence reads from the Short Read Archive (SRA; https://www.ncbi.nlm.nih.gov/sra, accession number; PRJNA144055) were mapped to *A. flavus* genomic region using Bowtie [60] and TopHat [61]. Fragment per kilobase pair of exon model per million fragment mapped (FPKM) values were calculated using Cufflinks [62]. To identify the extra copies of genes in *A. flavus* genomes, iron permease (Ftr1) and ferrooxidoreductase (Fet3) genes from *A. flavus* NRRL3357 were downloaded from GenBank and used in the BLAST analysis. Functional analysis of the IUC was performed by using BLAST [63] and protein domains were identified with InterProScan [64,65]. Conserved domains were identified by comparing with NCBI’s Conserved Domain Database (CDD) [66], and single nucleotide polymorphisms (SNPs) were called against the reference IUC of *A. oryzae* RIB40 using MAQ [67]. Multiple alignments were performed with CLUSTALW [68]. Sequences of IUC of the initial five genotypes are deposited in GenBank with accession numbers: KY586947-KY586951. 

### 2.3. Neighbor-Net Network

In order to assess the extent of diversity in IUC among *A. flavus* genotypes, genetic diversity among 215 *A. flavus* genotypes (not including the 5 original isolates) from cotton and corn crops produced in Arizona and Texas was examined using previously identified simple sequence repeats (SSR) at 17 loci [69]. Amplicons containing the SSRs were scored with GeneMarker [70] from traces produced with an ABI Capillary Sequencer [71]. Genetic distance between genotypes was calculated across the 17 loci with the START2 program [72]. The distance matrix was analyzed drawn with the Neighbor-Net algorithm in SplitsTree v4.13.1 [73]. Edges were colored according to grouping of genotypes based on structures of IUC.

### 2.4. Phylogenetic Analysis

Phylogenetic analysis of the partial iron permease gene (637-bp) from the 5 original *A. flavus* isolates, *A. nomius* NRRL13137, *A. oryzae* RIB40, and *A. parasiticus* SU-1 was performed with the UPGMA algorithm using MEGA7 [74]. Multiple alignments were generated with CLUSTALW. Data sets were bootstrapped with 1000 replicates to generate branch confidence values, and bootstrap values <80% were omitted. Any gaps in the sequence were treated as missing data, and no out-group was applied to build the phylogeny. 

### 2.5. PCR Profiling of the IUC

Whole genome sequencing of *A. flavus* isolates was done as previously described (Adhikari et al., 2016). Briefly, genomic DNA was isolated from conidia collected from a culture grown for 7 days (31 °C, dark) on 5/2 agar (5% V-8 vegetable juice, 2% salt, and 2% agar). The FastDNA SPIN Kit and the FastPrep Instrument were used following the manufacturer’s instructions (MP Biomedicals LLC, Santa Ana, CA, USA). Small DNA fragments and other contaminants were removed by applying DNA from the FastDNA SPIN Kit was applied to a SPIN filter column following manufacturer’s instructions. Quantification of genomic DNA was done by both spectrophotometer (modelND-1000, NanoDrop) and the Qubit dsDNA BR assay kit (Q32850) using the Qubit 1.0 Fluorometer (Thermo Fisher Scientific, Waltham, MA, USA) following the manufacturer’s guidelines. PCR validation was done by designing primers for each IUC gene (Supplementary Table S1) and using the PCR conditions described previously [45]. Deletions that were predicted previously by DELLY were further validated by PCR, using the primers that either bridge the putative deletion or amplify flanking regions.

## 3. Results

### 3.1. Identification, Characterization, and Validation of IUC

**Figure 1:**
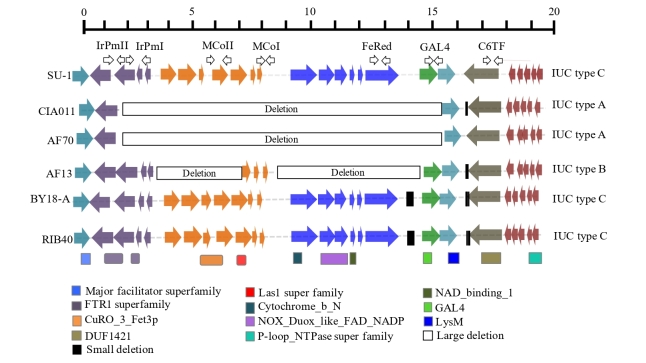
Iron utilization gene clusters from *Aspergillus flavus* (AF13, AF70, BY18-A, and CIA011), *Aspergillus parasiticus* (SU-1), and *Aspergillus oryzae* (RIB40). Top bar is a size reference (0 to 20 kb). Each gene is represented by a different color with arrows indicating direction of transcription and breaks indicating introns. The black and empty boxes indicate deletions. Open arrows indicate primer-binding sites and direction of amplification for primers used to assess the distribution of genes within *A. flavus* communities and letters above the arrows indicate primer names.

In order to identify the structural variants in the five initial *A. flavus* genotypes, genome sequences were compared with *A. oryzae* RIB40 reference [56]. Of the several regions with identified deletions, a 15 Kb region was highly variable among *A. flavus* isolates. The identified region contains 6 genes. Multiple alignments of the gene region show the location in a region homologous to the centromeric end of Superscaffold124 from *A. oryzae* RIB40. Genome-wide alignment indicates presence of only one such group of genes in the five initial *A. flavus* isolates, *A. oryzae*, and *A. nomius* while *A. fumigatus*, *A. nidulans*, and *A. niger* had no region with significant similarity. Functional analysis of the genes with BLAST and InterProScan indicated 6 genes (Figure 1, Table 2). Gene 1 on the centromeric-end is present in all isolates and is predicted to be a hypothetical protein. Gene 2, a LysM domain containing protein, and Gene 3, a C6 transcription factor containing a GAL4 domain, are present in only two of the 5 *A. flavus* (BY18-A and DO114-A). Genes 1 through 3 have the closest homologs in *A. oryzae* RIB40. Gene 4 is a ferric reductase gene with a ferric reductase-like transmembrane component also found in *A. oryzae* RIB40. Gene 5 encodes a multicopper oxidase with ferroxidation activity, which is the closest homolog (e-value = 4 × 10^−169^) of *fet3* gene from *A. oryzae* RIB40. A comparison of the predicted amino acid sequences from *A. flavus* showed >95% similarity with *fet3* from *A. oryzae* RIB40. The last gene (Gene 6) is an iron permease, which is the closest homolog to *ftrA* gene from *A. oryzae* RIB40 and has 95% similarity in predicted amino acid sequences to FtrA (Table 2). Search of iron permease (*ftr1*) and ferrooxidoreductase (*fet3*) genes in *A. flavus* genomes showed presence of multiple *ftr1* and *fet3* genes in few *A. flavus* isolates outside the IUC. Few of the *A. flavus* isolates with complete IUC have *ftr1* and *fet3* homologs outside the IUC. *A. flavus* isolates with either partial or complete deletion of IUC, however, either lack the extra copies of genes or have partially deleted genes outside the IUC. Interestingly, none of the *fet3* genes present outside the IUC were clustered with *ftr1* genes as seen in IUC. Expression analysis of the genes within IUC showed that all six genes are expressed. FPKM values varied between genes, with the second set of 3 genes having higher expression values than the first set of 3 genes (Supplementary Table S2). To validate the genes, all six were PCR amplified from genomic DNA extracted from the 5 initial *A. flavus* isolates. BY18-A and DO114-A have all IUC genes intact, while CIA011 and EC69-E are missing the middle two genes (gene 3 and 4) and have lost portions of the last two genes through deletion. 

### 3.2. Polymorphisms within IUC among A. flavus Isolates

**Figure 2:**
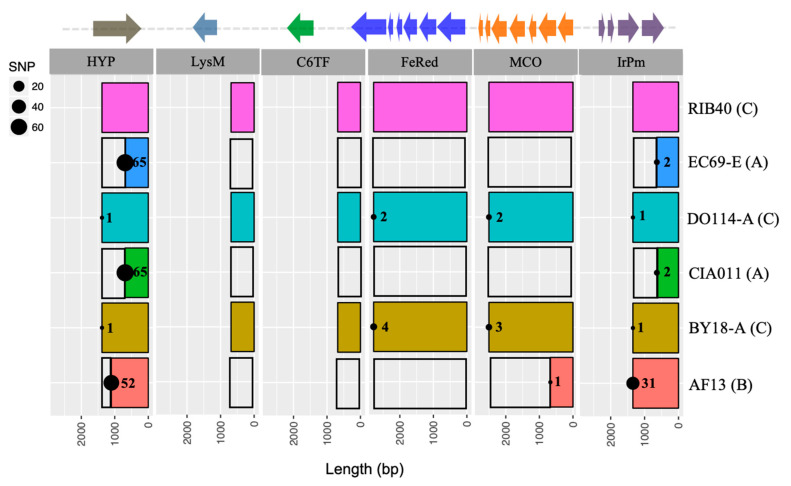
Iron utilization gene clusters of several *Aspergillus flavus* isolates with deletions and single nucleotide polymorphisms (SNPs) illustrated. The top bar illustrates the iron utilization gene cluster of *Aspergillus oryzae* RIB40. Arrows indicate direction of transcription and gene size. Numbers in bar segments indicate number of SNPs with reference to *A. oryzae* RIB40. HYP, Hypothetical protein; LysM, LysM domain containing protein; C6TF, C6 transcription factor; FeRed, Ferric reductase; MCO, Multicopper oxidase; IrPm, Iron permease.

Comparative analysis of the IUC from *A. flavus* isolates with reference *A. oryzae* RIB40 shows high levels of polymorphism (Figure 1). CIA011 and EC69-E have the smallest IUC with >12.5 kb deleted and only portions of genes 1 and 6 maintained (IUC type A). Both isolates have the same deletion with identical flanking sequences. AF13, which has two deletions (9 kb and 2 kb), has completely lost three genes (LysM domain containing protein, C6 transcription factor, and ferric reductase) and partially lost the multicopper oxidase (IUC type B). The iron permease gene in AF13 remains intact. The remaining two isolates (BY18-A and DO114-A) have complete IUCs with all 6 genes intact (IUC type C). Mapping of *A. flavus* IUCs to the corresponding *A. oryzae* IUC shows high levels of polymorphism in the gene that codes for the hypothetical protein. Isolates in IUC type A have the greatest density of SNPs (65), followed by isolates in IUC type B (52) (Figure 1). Iron permease genes from BY18-A, CIA011, DO114-A, and EC69-E are almost identical to the genes in *A. oryzae* RIB40, with only 1-2 SNPs. The *A. flavus* AF13 iron permease gene is highly divergent, with 31 SNPs (Figure 2). 

### 3.3. Production of Siderophores

**Figure 3:**
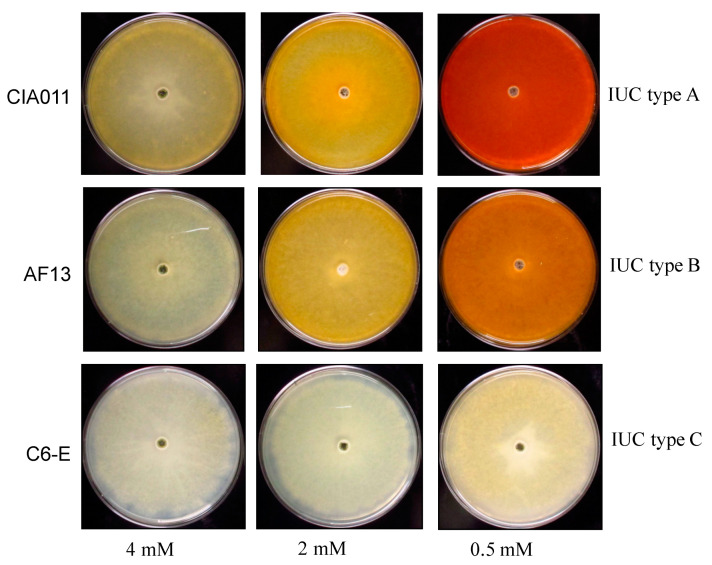
Effect of iron concentration on siderophore production by *Aspergillus flavus* genotypes. The orange color is due to the binding of the aspergillic acid with ferric ions from ferric ammonium citrate used as the iron source. Fungi were grown in Czapek’s agar with higher (4 mM), normal (2 mM), and lower (0.5 mM) ferric ammonium citrate (FAC) concentration.

Members of *Aspergillus* section Flavi produced a distinct bright orange color on the reverse of colonies grown on AFPA medium. The orange color results from reaction of ferric citrate with aspergillic acid, forming a colored complex [75]. All five of the initial *A. flavus* isolates used produced the characteristic orange color (Figure 3). Aspergillic acid, an N-hydroxylated pyrazine, is a naturally occurring hydroxamic siderophore [76]. A total of fifteen isolates (including the original five isolates) were tested, and similar results were obtained with all fifteen isolates grown on AFPA medium. Production of the characteristic orange color is used for taxonomic assessments [54] and indicates the ability to produce siderophores. 

### 3.4. Response to Reduced Iron Media

The response of *A. flavus* isolates to reduced iron media was variable and dependent upon IUC composition. Isolates categorized as IUC type C (e.g., C6-E) did not produce siderophores, as indicated by lack of orange color (aspergillic acid) on the reverse of the plate (Figure 3). On the other hand, *A. flavus* isolates with IUC types A and B produced more siderophores than IUC type C in media with higher concentration of FAC. IUC type C isolates grown on 0 mM FAC produced no siderophore, while those from IUC types A and B produced large quantities of siderophore, as shown by a bright orange color on the reverse of the plate (Figure 3). Increasing the concentration of FAC decreased siderophore production, and at 4 mM FAC concentration, siderophore production was greatly reduced. Measurement of red pixel count on the images of the reverse side of the plates showed higher number at both 0.5 mM and 2 mM concentrations as compared to 4 mM concentration for both isolates with partial or deleted IUC (Supplementary Table S3). The red pixel count is higher for isolate with partial or deleted IUC as compared to complete IUC. 

### 3.5. Presence of Reductive Iron Assimilation (RIA) Pathway

Fungi use secreted iron chelators (siderophores) and reductive iron assimilation (RIA) mechanisms to acquire iron in a high affinity manner. RIA processes originally characterized in *Saccharomyces cerevisiae* [77] involved three different genes. Ferric iron is reduced by ferric reductase and then oxidized by the iron multicopper oxidase while being transported across the plasma membrane by the high-affinity iron permease. All three genes (ferric reductase, multicopper oxidase, and iron permease) are located on one scaffold of the *A. flavus* genome assembly and separated by intergenic spaces of 1300-bp and 618-bp respectively (Figure 1). In isolates with IUC type A, both ferric reductase and *fet3* genes are missing and a partial *ftr1* gene is present (Table 3). *ftr1* gene remnants in all isolates with IUC type A have identical sequence ends. IUC type B isolates lack ferric reductase but have a complete *ftr1* gene with partial *fet3*. IUC type B isolates have remnants of various deletion events and are separated by the highest number of SNPs when compared with *A. oryzae* RIB40 or with each other (Figure 2).

### 3.6. Lineage-Specific Loss of IUC

**Figure 4:**
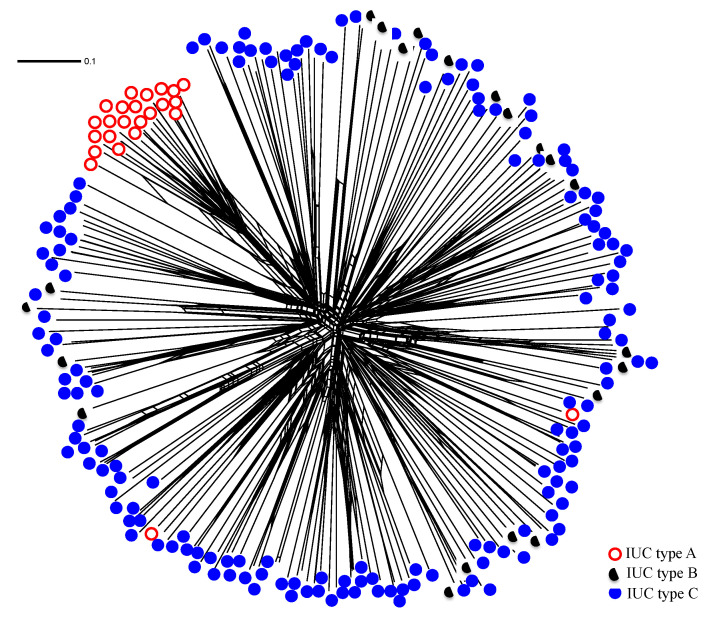
Neighbor-Net network for 215 *Aspergillus flavus* genotypes from Texas and Arizona. Network was generated by the split decomposition algorithm with allelic data from 17 SSR loci using SplitsTree4. Filled, half-filled, and empty circles represent genotypes with cluster type C, cluster type B, and cluster type A (Figure 1), respectively.

Relationships among 215 genotypes of *A. flavus* were analyzed using Neighbor-Net network based on genetic distance estimated from SSR data from 17 loci distributed across 8 chromosomes of *A. flavus* [69]. Initial examination revealed that 79% of the 215 genotypes have IUC type C, 10% have IUC type B, and 11% have IUC type A (Table 3). In general, the Neighbor-Net network reflects IUC structure (Figure 4). One well-resolved clade contains only genotypes with IUC type A with 81% of type A genotypes. Ten clades with 6 to 16 members contain IUC type C. Genotypes with IUC type B didn’t group into large clades but were co-distributed with IUC type C genotypes in many smaller clades. For IUC types B and C, deletion locations and sizes often vary, as do sequences of flanking regions. When genotypes are divided into groups based on the sequence flanking the IUC deletion, IUC type B segregates into three distinct groups while IUC type A and C each belong to a single group (Table 3).

### 3.7. Evolutionary Relationship of Iron Permease Gene

**Figure 5:**
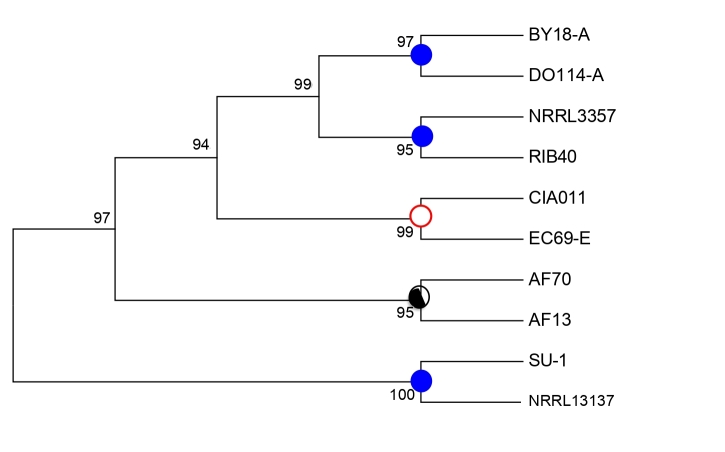
Phylogeny of partial iron permease gene (637-bp) from *Aspergillus flavus* isolates, *A. nomius* NRRL13137, *A. oryzae* RIB40, and *A. parasiticus* SU-1 using UPGMA algorithm. Supporting values for nodes were estimated with 1,000 bootstrap replicates. The phylogenetic tree lends support to lineage-specific loss of IUC as shown by Neighbor-Net analysis. Filled, partial, and empty circles on the nodes denote full, partial, and deleted, iron utilization gene clusters.

*Aspergillus flavus* isolates with IUC type C have iron permease genes with 1345-bp while isolates with deletions have partial *ftr1* with 637-bp. Phylogenetic analysis of these 637-bp from *A. flavus* isolates, *A. nomius* NRRL13137, *A. oryzae* RIB40, and *A. parasiticus* SU-1 resulted in a UPGMA tree with 5 well-supported clades (Figure 5). *A. parasiticus* SU-1 and *A. nomius* formed a clade separate from *A. flavus*. *A. flavus* isolates with IUC type B (AF13) and IUC type A (CIA011 and EC69-E) were resolved into a clade distinct from *A. oryzae* and *A. flavus* isolates with IUC type C. Phylogenetic relationships based on the 637-bp of iron permease gene recapitulate the grouping of the fungi based on the structure and presence/absence of genes in the IUC (Figure 2). A summary of the fungal isolates and the IUC type they belong to is provided in Supplementary Table S4. 

### 3.8. Presence of IUC in Other Fungi 

We searched genome sequence databases both within and outside *Aspergillus* to find homologues of the IUC. BLAST searches revealed that the IUC of *A. flavus* has very high similarity to *A. oryzae* RIB40 IUC (evalue = 0.00) (Table 2) followed by *A. flavus* NRRL3357. Recently sequenced *A. flavus* NRRL335 [78] and two other members of section *Flavi*, *A. nomius*, and *A. parasiticus*, both contain similar gene groups. When searched against larger datasets, except for *A. nidulans*, most *Aspergillus* genomes have at least one *ftrA*-*fetC* (Iron permease-ferroxidase) gene pair similar to the one found in *A. flavus* IUC [34]. The co-occurrence of the iron permease/multicopper oxidase genes has also been reported in many fungal genomes outside *Aspergillus.* For example, genes similar to *fet3* and *ftr1* have counterparts in many Ascomycete (*Neofusicoccum parvum*, evalue=4× 10^−116^; *Fusarium oxysporum*, evalue = 5 × 10^−107^) and Basidiomycete (*Ustilago maydis*, evalue = 1× 10^−125^) fungi [8,79].

## 4. Discussion

Iron uptake and homeostasis functions are important for pathogenesis, and iron levels influence growth and development of *A. flavus*. There is considerable genetic diversity among isolates of *A. flavus*, and this variation is particularly frequent in genes encoding products used to produce secondary metabolites. In the current study, we applied whole genome comparative analysis to assess diversity among *A. flavus* isolates. In doing so, we identified group of genes potentially functional in iron uptake and utilization and forming a putative gene cluster (IUC) spanning 15 Kb. The putative IUC identified in this work contains 6 genes, including putative transcription factors. Among the various *A. flavus* isolates, multiple indels that disable genes in the IUC were detected in multiple genotypes, suggesting these mutations were fixed in the resulting clonal lineage. The current work indicates that *A. flavus* may have two different mechanisms for iron utilization, a siderophore-mediated iron utilization system and a reductive iron assimilation (RIA) pathway. Sequence similarity to genes responsible for high-affinity iron uptake in other fungi suggests a fully functional IUC in most *A. flavus* genotypes. Isolates missing genes in the IUC responded differently to iron starvation conditions by possibly employing a different pathway for iron uptake and utilization. These isolates produced siderophores when grown under low-iron conditions, while those with a complete IUC did not produce siderophores (Figure 3). These results, together with the *A. flavus* population profiling data, indicate lineage-specific loss of iron utilization genes and suggest the possibility that different genotypes of *A. flavus* have distinct iron uptake and utilization systems. 

The IUC identified in the current study resides on a region homologous to Chromosome 5 of *A. oryzae* RIB40 and primarily contains genes with predicted functions in iron uptake and utilization (Figure 1). Functional prediction of IUC genes indicates possible involvement in the uptake and utilization of iron (Table 2). The first two genes are hypothetical proteins with either a GAL4 domain or a fungal-specific LysM domain. LysM domain containing proteins act as effectors that play important roles in promoting virulence [80]. The third gene in the IUC contains a domain of unknown function with homology to fungal C6 transcription factors. Some members of the fungal-specific C6 transcription factor family are strongly associated with secondary metabolite gene clusters in *A. flavus* and *A. parasiticus* [81]. For example, *aflR*, one of the C6 transcription factors in *A. flavus,* is the key positive regulator of expression of most aflatoxin genes [82,83]. Based on the position of the gene in the IUC, we anticipate that this C6 transcription factor plays a role in the regulation of genes involved in iron uptake and utilization. The IUC ferric reductase gene has a ferric reductase like transmembrane domain. Ferric reductase facilitates the use of siderophore-bound iron. Ferric reductases in fungi play important roles in iron acquisition and virulence [84]. Another gene of particular interest is *fet3*, encoding the multicopper oxidase (MCO). Based on the domain structure of the predicted Fet3 protein, we anticipate this enzyme is involved in the ferroxidation process (Figure 1). MCO plays an important role in high affinity iron uptake through Fe^2+^ to Fe^3+^ [85,86] conversion. The gene next to MCO in the IUC is a putative iron permease, involved in iron transport in iron-depleted environments. In many pathogenic fungi [8,87] products of similar iron permease genes are virulence factors. Clustering of genes encoding MCO and an iron permease is not unique to *A. flavus*. Such clustering is also found in many other fungi [27,88]. Although the link between ferroxidation and iron transport by the iron permeation depends on the coupling of these two enzymes (iron permease and multicopper oxidase), the process is not well understood [8]. The current results suggest reductive mobilization of iron is common in *A. flavus*.

The complete IUC was conserved in most (79%) *A. flavus* isolates analyzed in this study. Considering the wide distribution of complete IUC among *A. flavus* genotypes, RIA can be considered the index (basal) pathway for high affinity iron uptake [8]. RIA is an extracellular membrane-bound process whereby ferric iron is reduced and subsequently oxidized while being transported across the plasma membrane by the high-affinity iron permease Ftr1 [8,89]. As observed in *A. flavus* (Figure 1), Ftr1 and Fet3 are closely linked and co-dependent on the plasma membrane [25,90]. In *S. cerevisiae* vacuoles store iron, and homologs of Fet3/Ftr1 are reported to be involved in vacuolar iron storage in addition to their involvement in iron uptake [91,92,93]. Membrane-bound reductive iron assimilatory systems have been reported for a broad array of fungi [8,27,28,29,30,31,32,94] but the existence of this system in *A. flavus* was previously unknown. Clustering of the genes in biosynthetic pathways is the hallmark of bacteria and filamentous fungi [95]. In that sense the potential clustering of the IUC genes of *A. flavus* is not surprising, but the magnitude of differences among closely related *A. flavus* genotypes reveals unexpected diversity within species of *A. flavus* in terms of iron uptake and utilization. 

Discovery of new metabolic pathways and associated gene clusters through genome mining is greatly accelerated by the availability of sequenced genomes from multiple species of *Aspergillus* [96,97,98]. The genes identified within IUC and involved in iron ferroxidation and permeation have homologs in other fungi [79]. Homologs of *fet3* and *ftr1* genes were identified from *A. flavus* genomes but in our case none of those genes seem to be clustered outside the IUC. This suggests that fungal genera share common iron uptake utilization pathways, including genes involved in ferroxidation and permeation, siderophore synthesis, and transport. However, differences may exist in mechanisms through which iron utilization could be modulated by environmental factors. Phylogenetic analysis based on the partial iron permease gene (Figure 5) reflects evolution of the IUC in *Aspergillus* section *Flavi* and strongly supports the relationship observed by multiple alignment of the IUC. The diversity of the IUC among *A. flavus* isolates suggest that *A. flavus* has iron utilization pathways similar to those observed in many other fungi and that these pathways could play important roles in adaptation to the diverse ecological niches this species is known to occupy. 

To assess the extent of diversity in IUC among *A. flavus* genotypes, we characterized the patterns of genetic variation among genotypes from Arizona and Texas. A total of 215 genotypes representing *A. flavus* associated with either cotton or corn were profiled. The structure of the Neighbor-Net network largely reflects IUC structure (Figure 4), with clades including only genotypes lacking copies of all the IUC genes or only genotypes with all the IUC genes intact. Complete IUC deletions have common boarder sequences (Table 3), suggesting an ancient deletion event that has been retained through clonal evolution over tens of thousands of years. In contrast to the homogeneity in this clade, those containing partial IUCs have more diversity (Figure 4, Table 3). Sequencing of flanking regions from genotypes with partial IUC revealed a diversity of flanking sequences reflecting multiple deletion events (Table 3). In some cases, the same deletion occurred in multiple clades. This may have resulted from incomplete lineage sorting or gene flow. Repeated loss of IUC function and success of lineages where the IUC has been lost may suggest an adaptive advantage to this loss. Although specific ecological or environmental factors contributing to genetic differences among *A. flavus* genotypes are not known, soil types differing in iron content could have contributed to this differential adaptation. This pattern of multiple deletion events has been demonstrated for other biosynthetic gene clusters [52,99,100,101] in *Aspergillus* section *Flavi*. 

Under iron-limited conditions, most fungi synthesize and secrete siderophores, which are small organic compounds that bind ferric iron with high affinity and specificity [10]. Virtually all fungi express a nonreductive uptake system that is specific for siderophore iron chelates [102]. Our analysis revealed that *A. flavus* isolates with IUC type C are equipped with all genes required for an RIA system, while isolates with IUC type A and B rely on siderophore-mediated iron uptake system. To show the function of the IUC type identified in this study on iron utilization in *A. flavus*, isolates with different deletions in IUC were exposed to varying iron concentrations (Figure 3). When grown under iron-limited conditions, *A. flavus* isolates without IUC produced large quantities of siderophores indicating siderophore-mediated iron uptake and utilization. In contrast, isolates with complete IUC did not produce siderophores even under iron-limited conditions, suggesting their lack of reliance on siderophore-mediated iron uptake system. Nevertheless, these isolates have retained capacity to produce siderophores, as demonstrated by production on AFPA. It has been shown that deprivation of iron results in increased synthesis of siderophores in *E. coli* [103]; similar production of siderophores under iron-limited conditions in *A. flavus* suggests an analogous function. Although the genetic mechanisms that shape iron utilization pathways are not fully understood, one explanation for the observed diversity is that *A. flavus*, as an animal and plant pathogen, evolved in an environment that was populated by many other microorganisms. Diversity of uptake systems could have enabled *A. flavus* to effectively compete with other microorganisms for the limited amounts of available iron in the environment while facilitating facultative pathogenicity to plants and animals. Global climate change will influence not only the quantity of crops available but also compositions of edible crop components including reduction in iron content [104]. Competition for iron may be increasingly important to the ability of atoxigenic biocontrol strains of *A. flavus* to outcompete aflatoxin producers. The variability in iron acquisition processes described in the current work should be considered when selecting atoxigenic strains for aflatoxin management in altered climates.

Analysis of diversity and evolution within *Aspergillus* section *Flavi* suggests the possibility that *A. flavus* may have multiple pathways for iron acquisition and that the IUC has been modified in *A. flavus* lineages through multiple deletion events. Certain IUC deletions have been retained during lineage divergence. A component of the *A. flavus* RIA, the ferroxidation/permeation iron uptake system, shows similarities with other fungi where it is important for virulence [105,106,107]. One of the intriguing questions is why there is variation among *A. flavus* genotypes in iron acquisition genes despite evolutionary closeness. It is possible that the adaptation of *A. flavus* to different conditions may ultimately determine the manner in which iron is acquired from the environment. However, empirical data is required to test this hypothesis. Furthermore, *A. flavus* is the second leading cause of invasive aspergillosis and the genes identified in this study could be useful as targets for disease monitoring and management. Improved understanding of iron uptake and utilization might facilitate better understanding and management of aflatoxin contamination of food and feed.

## Figures and Tables

**Table 1:** *Aspergillus flavus* isolates for which sequence analyses of the iron utilization gene cluster were performed.

Isolate	Substrate	Aflatoxin *	Culture Accession/Source	Reference
AF13	Cotton	Toxigenic	ATCC 96044	[53]
BY18-A	Maize	Atoxigenic	USDA-ARS, Tucson	[52]
CIA011	Maize	Atoxigenic	USDA-ARS, Tucson	[52]
DO114-A	Maize	Atoxigenic	USDA-ARS, Tucson	[52]
EC69-E	Maize	Atoxigenic	USDA-ARS, Tucson	[52]

* Ability to produce aflatoxin.

**Table 2:** Annotation of putative iron utilization gene cluster.

Protein ID	Length ^¶^	Homologue ^$^	Nucleotide Identity (%), Coverage (%)	Functional annotation ^£^	Protein Domain ^§^
BAE62583.1	459	Hypothetical protein, *Aspergillus oryzae* RIB40	96, 99	C6 transcription factor	DUF1421
BAE62584.1	229	Hypothetical protein, *Aspergillus oryzae* RIB40	99, 86	Predicted protein	LysM
BAE62585.1	225	Hypothetical protein, *Aspergillus oryzae* RIB40	99, 98	Unnamed protein	GAL4
BAE62586.1	825	Ferric reductase, *Aspergillus oryzae* RIB40	94, 93	Ferric reductase	NOX_Duox_like NAD_NADP, NAD_binding_1
BAE62587.1	621	Ferrooxidoreductase, *Aspergillus oryzae* RIB40	89, 69	Multicopper oxidase	CuRO_1_Fet3p, Las1 and Cupredoxin superfamily
BAE62588.1	370	Iron permease, *Aspergillus oryzae* RIB40	91, 82	Iron permease	Iron permease Ftr1 family

^¶^ Amino acid length. ^$^ Origin of most closely related protein homologue. ^£^ Predicted function based on BLAST against UniProt database. ^§^ Protein domain predicted by InterProScan [65] and Conserved Domain Database [66].

**Table 3:** Characterization of deletions in the iron utilization gene clusters from *Aspergillus flavus* isolates.

Cluster Type ^§^	Genes Deleted ^¶^	Sequence End (5′-3′) ^⌘^	Sequence End (3′-5′)	Group ^∞^	Proportion of Isolates ^$^
Type C	None	CGGGCAACTCTCCCCTTGCG	TATGTTCCAGTCAAAGACGT	I	79%
Type B	FeRed, MCO	CTGGCAGTTCATCGTTTGCG	TATGTTCCAGTCAAAGACGT	II	1%
Type A	IrPm (partial), MCO, FeRed, GAL4	TGTCCTATCTATCTCTGGTG	CTGGTGTTGCAGTAGGCTAT	III	11%
Type B	IrPm (partial), MCO, FeRed, GAL4, LysM	TGTCCTATCTATCTCTGGTG	CTGGTGTTGCAGTAGGCTAT	II	1%
Type B	FeRed, MCO (partial)	CTGGCAGTTCTTCGTTTGCG	T-TGATCATGCTGCGGAAGG	IV	2%
Type B	FeRed, MCO (partial)	CTGGCAGTTCATCTCTTGCG	T-TGATCATGCTGCGGGAGG	V	6%

^§^ Cluster type based on the structure of the cluster. ^¶^ Genes deleted from the IUC. FeRed, Ferric reductase; IrPm, Iron permease; MCO, Multicopper oxidase; GAL4, GAL4 domain containing protein; LysM, LysM domain containing protein. ^⌘^ Ends of the sequences flanking the same deletion or region corresponding to flanking region. ^∞^ Grouping of the isolates based on genes deleted and sequenced ends. Isolates in the same group have similar deletion and sequence ends. ^$^ Proportion based on genomic profiling of 215 Texas and Arizona *A. flavus* genotypes.

## Data Availability

Sequences of the IUC of the initial five genotypes are deposited in GenBank with accession numbers: KY586947- KY586951.

## Supplementary Materials

The following are available online at https://www.mdpi.com/2076-2607/9/1/137/s1, Table S1: Primers used to amplify genes in the iron utilization gene cluster, Table S2: Expression profiling of *Aspergillus flavus* genes within IUC, Table S3: Count of the red pixel in the images of Czapek’s agar plates with different concentrations of FAC. Table S4: List of the *Aspergillus flavus*, *A. nomius*, *A. oryzae*, and *A. parasiticus* strains used in the analysis and the IUC type they belong to.

## References

[1] Haber F., Weiss J. (1934). The catalytic decomposition of hydrogen peroxide by iron salts. Proc. R. Soc. Lond..

[2] Halliwell B., Gutteridge J.M. (1984). Role of iron in oxygen radical reactions. Methods Enzymol..

[3] Kehrer J.P. (2000). The Haber–Weiss reaction and mechanisms of toxicity. Toxicology.

[4] Andrews N.C. (2008). Forging a field: The golden age of iron biology. Blood.

[5] De Domenico I., Ward D.M.V., Kaplan J. (2008). Regulation of iron acquisition and storage: Consequences for iron-linked disorders. Nat. Rev. Mol. Cell Biol..

[6] Schrettl M., Beckmann N., Varga J., Heinekamp T., Jacobsen I.D., Jöchl C., Moussa T.A., Wang S., Gsaller F., Blatzer M. (2010). HapX-Mediated Adaption to Iron Starvation Is Crucial for Virulence of Aspergillus fumigatus. PLoS Pathog..

[7] Weinberg E.D. (1999). The role of iron in protozoan and fungal infectious diseases. J. Eukaryot. Microbiol..

[8] Kosman D.J. (2003). Molecular mechanisms of iron uptake in fungi. Mol. Microbiol..

[9] Guerinot M.L., Meidl E.J., Plessner O. (1990). Citrate as a siderophore in Bradyrhizobium japonicum. J. Bacteriol..

[10] Neilands J.B. (1995). Siderophores: Structure and Function of Microbial Iron Transport Compounds. J. Biol. Chem..

[11] Haas H., Eisendle M., Turgeon B.G. (2008). Siderophores in Fungal Physiology and Virulence. Annu. Rev. Phytopathol..

[12] Haas H. (2003). Molecular genetics of fungal siderophore biosynthesis and uptake: The role of siderophores in iron uptake and storage. Appl. Microbiol. Biotechnol..

[13] Matzanke B.F., Bill E., Trautwein A.X., Winkelmann G. (1987). Role of siderophores in iron storage in spores of Neurospora crassa and Aspergillus ochraceus. J. Bacteriol..

[14] Schrettl M., Kim H.S., Eisendle M., Kragl C., Nierman W.C., Heinekamp T., Werner E.R., Jacobsen I., Illmer P., Yi H. (2008). SreA-mediated iron regulation in Aspergillus fumigatus. Mol. Microbiol..

[15] Jung W.H., Kronstad J.W. (2008). Iron and fungal pathogenesis: A case study with Cryptococcus neoformans. Cell. Microbiol..

[16] Nevitt T., Thiele D.J. (2011). Host Iron Withholding Demands Siderophore Utilization for Candida glabrata to Survive Macrophage Killing. PLoS Pathog..

[17] Philpott C.C., Protchenko O. (2008). Response to Iron Deprivation in Saccharomyces cerevisiae. Eukaryot. Cell.

[18] Schrettl M., Bignell E., Kragl C., Joechl C., Rogers T., Arst H.N., Haynes K., Haas H. (2004). Siderophore Biosynthesis But Not Reductive Iron Assimilation Is Essential for Aspergillus fumigatus Virulence. J. Exp. Med..

[19] Caza M., Kronstad J.W. (2013). Shared and distinct mechanisms of iron acquisition by bacterial and fungal pathogens of humans. Front. Cell. Infect. Microbiol..

[20] Howard D.H. (1999). Acquisition, Transport, and Storage of Iron by Pathogenic Fungi. Clin. Microbiol. Rev..

[21] Morrissey J., Guerinot M.L. (2009). Iron Uptake and Transport in Plants: The Good, the Bad, and the Ionome. Chem. Rev..

[22] Philpott C.C. (2006). Iron uptake in fungi: A system for every source. Biochim. Biophys. Acta.

[23] Ardon O., Nudelman R., Caris C., Libman J., Shanzer A., Chen Y., Hadar Y. (1998). Iron Uptake in Ustilago maydis: Tracking the Iron Path. J. Bacteriol..

[24] Dancis A., Yuan D.S., Haile D., Askwith C., Eide D., Moehle C., Kaplan J., Klausner R.D. (1994). Molecular characterization of a copper transport protein in S. cerevisiae: An unexpected role for copper in iron transport. Cell.

[25] Stearman R., Yuan D.S., Yamaguchi-Iwai Y., Klausner R.D., Dancis A. (1996). A Permease-Oxidase Complex Involved in High-Affinity Iron Uptake in Yeast. Science.

[26] Heymann P., Ernst J.F., Winkelmann G., Winkelmann G. (2000). Identification and substrate specificity of a ferrichrome-type siderophore transporter (Arn1p) in Saccharomyces cerevisiae. FEMS Microbiol. Lett..

[27] Askwith C., Kaplan J. (1997). An Oxidase-Permease-based Iron Transport System inSchizosaccharomyces pombeand Its Expression inSaccharomyces cerevisiae. J. Biol. Chem..

[28] Ecker D.J., Emery T. (1983). Iron uptake from ferrichrome A and iron citrate in Ustilago sphaerogena. J. Bacteriol..

[29] Fedorovich D., Protchenko O., Lesuisse E. (1999). Iron uptake by the yeast Pichia guilliermondii. Flavinogenesis and reductive iron assimilation are co-regulated processes. BioMetals.

[30] Lesuisse E., Casterassimon M., Labbé P. (1995). Ferrireductase Activity in Saccharomyces cerevisiae and Other Fungi: Colorimetric Assays on Agar Plates. Anal. Biochem..

[31] Nyhus K.J., Jacobson E.S. (1999). Genetic and Physiologic Characterization of Ferric/Cupric Reductase Constitutive Mutants of Cryptococcus neoformans. Infect. Immun..

[32] Timmerman M.M., Woods J.P. (1999). Ferric Reduction Is a Potential Iron Acquisition Mechanism for Histoplasma capsulatum. Infect. Immun..

[33] Franken A.C.W., Lechner B.E., Werner E.R., Haas H., Lokman B.C., Ram A.F.J., Van den Hondel C.A., De Weert S., Punt P.J. (2014). Genome mining and functional genomics for siderophore production in Aspergillus niger. Brief. Funct. Genom..

[34] Haas H. (2012). Iron—A Key Nexus in the Virulence of Aspergillus fumigatus. Front. Microbiol..

[35] Schrettl M., Haas H. (2011). Iron homeostasis—Achilles’ heel of Aspergillus fumigatus?. Curr. Opin. Microbiol..

[36] Eisendle M., Oberegger H., Zadra I., Haas H. (2003). The siderophore system is essential for viability of Aspergillus nidulans: Functional analysis of two genes encoding l-ornithine N 5-monooxygenase (sidA) and a non-ribosomal peptide synthetase (sidC). Mol. Microbiol..

[37] Haas H., Zadra I., Stöffler G., Angermayr K. (1999). TheAspergillus nidulansGATA Factor SREA Is Involved in Regulation of Siderophore Biosynthesis and Control of Iron Uptake. J. Biol. Chem..

[38] Oberegger H., Zadra I., Schoeser M., Abt B., Parson W., Haas H. (2002). Identification of members of the Aspergillus nidulans SREA regulon: Genes involved in siderophore biosynthesis and utilization. Biochem. Soc. Trans..

[39] Almeida R.S., Wilson D., Hube B. (2009). Candida albicans iron acquisition within the host. FEMS Yeast Res..

[40] Ratledge C., Dover L.G. (2000). Iron Metabolism in Pathogenic Bacteria. Annu. Rev. Microbiol..

[41] Khlangwiset P., Wu F. (2010). Costs and efficacy of public health interventions to reduce aflatoxin-induced human disease. Food Addit. Contam. Part A.

[42] Shephard G. (2008). Impact of mycotoxins on human health in developing countries. Food Addit. Contam. Part A.

[43] Probst C., Schulthess F., Cotty P.J. (2010). Impact ofAspergillussectionFlavicommunity structure on the development of lethal levels of aflatoxins in Kenyan maize (Zea mays). J. Appl. Microbiol..

[44] Mehl H.L., Cotty P.J. (2008). Variability in competitive ability among Aspergillus flavus vegetative compatibility groups during maize infection. Phytopathology.

[45] Mehl H.L., Cotty P.J. (2010). Variation in Competitive Ability among Isolates of Aspergillus flavus from Different Vegetative Compatibility Groups During Maize Infection. Phytopathology.

[46] Mehl H., Cotty P.J. (2011). Influence of the Host Contact Sequence on the Outcome of Competition amongAspergillus flavusIsolates during Host Tissue Invasion. Appl. Environ. Microbiol..

[47] Aziz N.H., Shahin A.A.M., Abou-Zeid A.A.M., El-Zeany S.A. (2000). Correlation of growth and aflatoxin production by Asper-gillus flavus with some essential metals in gamma irradiated crushed corn. Food/Nahrung.

[48] Cuero R., Ouellet T., Yu J., Mogongwa N. (2003). Metal ion enhancement of fungal growth, gene expression and aflatoxin synthesis in Aspergillus flavus: RT-PCR characterization. J. Appl. Microbiol..

[49] LaRochelle O., Gagné V., Charron J., Soh J.-W., Séguin C. (2001). Phosphorylation Is Involved in the Activation of Metal-regulatory Transcription Factor 1 in Response to Metal Ions. J. Biol. Chem..

[50] Cuero R., Ouellet T. (2005). Metal ions modulate gene expression and accumulation of the mycotoxins aflatoxin and zearalenone. J. Appl. Microbiol..

[51] Liu J., Sun L., Zhang N., Zhang J., Guo J., Li C., Rajput S.A., Qi D. (2016). Effects of Nutrients in Substrates of Different Grains on Aflatoxin B1 Production by Aspergillus flavus. Biomed. Res. Int..

[52] Adhikari B.N., Bandyopadhyay R., Cotty P.J. (2016). Degeneration of aflatoxin gene clusters in Aspergillus flavus from Africa and North America. AMB Express.

[53] Cotty P.J. (1989). Virulence and Cultural Characteristics of TwoAspergillus flavusStrains Pathogenic on Cotton. Phytopathology.

[54] Pitt J., Hocking A.D., Glenn D.R. (1983). An improved medium for the detection ofAspergillus flavusandA. parasiticus. J. Appl. Bacteriol..

[55] Abramoff M., Magalhaes P., Ram S. (2004). Image processing with ImageJ. Biophotonics Int..

[56] Machida M., Asai K., Sano M., Tanaka T., Kumagai T., Terai G., Kusumoto K.-I., Arima T., Akita O., Kashiwagi Y. (2005). Genome sequencing and analysis of Aspergillus oryzae. Nature.

[57] Rausch T., Zichner T., Schlattl A., Stütz A.M., Benes V., Korbel J.O. (2012). DELLY: Structural variant discovery by integrated paired-end and split-read analysis. Bioinformatics.

[58] Cantarel B.L., Korf I., Robb S.M., Parra G., Ross E., Moore B., Holt C., Alvarado A.S., Yandell M. (2008). MAKER: An easy-to-use annotation pipeline designed for emerging model organism genomes. Genome Res..

[59] Yu J., Fedorova N.D., Montalbano B.G., Bhatnagar D., Cleveland T.E., Bennett J.W., Nierman W.C. (2011). Tight control of mycotoxin biosynthesis gene expression in Aspergillus flavus by temperature as revealed by RNA-Seq. FEMS Microbiol. Lett..

[60] Langmead B., Trapnell C., Pop M., Salzberg S.L. (2009). Ultrafast and memory-efficient alignment of short DNA sequences to the human genome. Genome Biol..

[61] Trapnell C., Pachter L., Salzberg S.L. (2009). TopHat: Discovering splice junctions with RNA-Seq. Bioinformatics.

[62] Trapnell C., Williams B.A., Pertea G., Mortazavi A., Kwan G., Van Baren M.J., Salzberg S.L., Wold B.J., Pachter L. (2010). Transcript assembly and quantification by RNA-Seq reveals unannotated transcripts and isoform switching during cell differentiation. Nat. Biotechnol..

[63] Altschul S.F., Gish W., Miller W., Myers E.W., Lipman D.J. (1990). Basic local alignment search tool. J. Mol. Biol..

[64] Hunter S., Apweiler R., Attwood T.K., Bairoch A., Bateman A., Binns D., Bork P., Das U., Daugherty L., Duquenne L. (2009). InterPro: The integrative protein signature database. Nucleic Acids Res..

[65] Jones P., Binns D., Chang H.-Y., Fraser M., Li W., McAnulla C., McWilliam H., Maslen J., Mitchell A., Nuka G. (2014). InterProScan 5: Genome-scale protein function classification. Bioinform..

[66] Marchler-Bauer A., Anderson J.B., Derbyshire M.K., DeWeese-Scott C., Gonzales N.R., Gwadz M., Hao L., He S., Hurwitz D.I., Jackson J.D. (2007). CDD: A conserved domain database for interactive domain family analysis. Nucleic Acids Res..

[67] Li H., Ruan J., Durbin R. (2008). Mapping short DNA sequencing reads and calling variants using mapping quality scores. Genome Res..

[68] Thompson J.D., Higgins D.G., Gibson T.J. (1994). CLUSTAL W: Improving the sensitivity of progressive multiple sequence alignment through sequence weighting, position-specific gap penalties and weight matrix choice. Nucleic Acids Res..

[69] Grubisha L.C., Cotty P.J. (2009). Twenty-four microsatellite markers for the aflatoxin-producing fungusAspergillus flavus. Mol. Ecol. Resour..

[70] Holland M.M., Parson W. (2011). GeneMarker® HID: A Reliable Software Tool for the Analysis of Forensic STR Data. J. Forensic Sci..

[71] Grubisha L.C., Cotty P.J. (2010). Genetic isolation among sympatric vegetative compatibility groups of the aflatoxin-producing fungus Aspergillus flavus. Mol. Ecol..

[72] Jolley K., Feil E.J., Chan M.-S., Maiden M.C.J. (2001). Sequence type analysis and recombinational tests (START). Bioinformatics.

[73] Huson D.H., Bryant D. (2005). Application of Phylogenetic Networks in Evolutionary Studies. Mol. Biol. Evol..

[74] Kumar S., Tamura K., Nei M. (1994). MEGA: Molecular Evolutionary Genetics Analysis software for microcomputers. Comput. Appl. Biosci..

[75] Assante G., Camarda L., Locci R., Merlini L., Nasini G., Papadopoulos E. (1981). Isolation and structure of red pigments from Aspergillus flavus and related species, grown on a differential medium. J. Agric. Food Chem..

[76] Dutcher J.D. (1958). Aspergillic acid; an antibiotic substance produced by Aspergillus flavus. J. Biol. Chem..

[77] Lesuisse E., Labbe P. (1989). Reductive and Non-reductive Mechanisms of Iron Assimilation by the Yeast Saccharomyces cerevisiae. J. Gen. Microbiol..

[78] Nierman W.C., Yu J., Fedorova-Abrams N.D., Losada L., Cleveland T.E., Bhatnagar D., Bennett J.W., Dean R., Payne G.A. (2015). Genome Sequence of Aspergillus flavus NRRL 3357, a Strain That Causes Aflatoxin Contamination of Food and Feed. Genome Announc..

[79] Eichhorn H., Lessing F., Winterberg B., Schirawski J., Kämper J., Müller P., Kahmann R. (2006). A Ferroxidation/Permeation Iron Uptake System Is Required for Virulence in Ustilago maydis. Plant Cell.

[80] Kombrink A., Thomma B.P.H.J. (2013). LysM Effectors: Secreted Proteins Supporting Fungal Life. PLoS Pathog..

[81] Linz J.E., Wee J., Roze L.V. (2014). Aspergillus parasiticus SU-1 Genome Sequence, Predicted Chromosome Structure, and Comparative Gene Expression under Aflatoxin-Inducing Conditions: Evidence that Differential Expression Contributes to Species Phenotype. Eukaryot. Cell.

[82] Ehrlich K.C., Montalbano B.G., Cary J.W. (1999). Binding of the C6-zinc cluster protein, AFLR, to the promoters of aflatoxin pathway biosynthesis genes in Aspergillus parasiticus. Gene.

[83] Woloshuk C.P., Foutz K.R., Brewer J.F., Bhatnagar D., E Cleveland T., A Payne G. (1994). Molecular characterization of aflR, a regulatory locus for aflatoxin biosynthesis. Appl. Environ. Microbiol..

[84] Saikia S., Oliveira D., Hu G., Kronstad J.W. (2014). Role of Ferric Reductases in Iron Acquisition and Virulence in the Fungal Pathogen Cryptococcus neoformans. Infect. Immun..

[85] Tamayo-Ramos J.A., Barends S., Verhaert R.M.D., De Graaff L.H. (2011). The Aspergillus niger multicopper oxidase family: Analysis and overexpression of laccase-like encoding genes. Microb. Cell Fact..

[86] Kues U., Ruhl M. (2011). Multiple Multi-Copper Oxidase Gene Families in Basidiomycetes—What for?. Curr. Genom..

[87] Ibrahim A.S., Gebremariam T., Lin L., Luo G., Husseiny M.I., Skory C.D., Fu Y., French S.W., Jr J.E.E., Spellberg B. (2010). The high affinity iron permease is a key virulence factor required for Rhizopus oryzae pathogenesis. Mol. Microbiol..

[88] Lian T., Simmer M.I., D’Souza C.A., Steen B.R., Zuyderduyn S.D., Jones S.J.M., Marra M.A., Kronstad J.W. (2005). Iron-regulated transcription and capsule formation in the fungal pathogen Cryptococcus neoformans. Mol. Microbiol..

[89] Kosman D.J. (2010). Redox Cycling in Iron Uptake, Efflux, and Trafficking. J. Biol. Chem..

[90] Singh A., Severance S., Kaur N., Wiltsie W., Kosman D.J. (2006). Assembly, Activation, and Trafficking of the Fet3p·Ftr1p High Affinity Iron Permease Complex inSaccharomyces cerevisiae. J. Biol. Chem..

[91] Urbanowski J.L., Piper R.C. (1999). The Iron Transporter Fth1p Forms a Complex with the Fet5 Iron Oxidase and Resides on the Vacuolar Membrane. J. Biol. Chem..

[92] Singh A., Kaur N., Kosman D.J. (2007). The Metalloreductase Fre6p in Fe-Efflux from the Yeast Vacuole. J. Biol. Chem..

[93] Rees E.M., Thiele D.J. (2007). Identification of a Vacuole-associated Metalloreductase and Its Role in Ctr2-mediated Intracellular Copper Mobilization. J. Biol. Chem..

[94] Morrissey J.A., Williams P.H., Cashmore A.M. (1996). Candida Albicans has a Cell-Associated Ferric-Reductase Activity which is Regulated in Response to Levels of Iron and Copper. Microbiology.

[95] Osbourn A. (2010). Gene Clusters for Secondary Metabolic Pathways: An Emerging Theme in Plant Biology. Plant Physiol..

[96] Medema M.H., Blin K., Cimermancic P., De Jager V., Zakrzewski P., Fischbach M.A., Weber T., Takano E., Breitling R. (2011). antiSMASH: Rapid identification, annotation and analysis of secondary metabolite biosynthesis gene clusters in bacterial and fungal genome sequences. Nucleic Acids Res..

[97] Inglis D., Binkley J., Skrzypek M.S., Arnaud M.B., Cerqueira G.C., Shah P., Wymore F., Wortman J.R., Sherlock G. (2013). Comprehensive annotation of secondary metabolite biosynthetic genes and gene clusters of Aspergillus nidulans, A. fumigatus, A. niger and A. oryzae. BMC Microbiol..

[98] Andersen M.R., Nielsen J.B., Klitgaard A., Petersen L.M., Zachariasen M., Hansen T.J., Blicher L.H., Gotfredsen C.H., Larsen T.O., Nielsen K.F. (2012). Accurate prediction of secondary metabolite gene clusters in filamentous fungi. Proc. Natl. Acad. Sci. USA.

[99] Chang P.-K., Abbas H.K., Weaver M.A., Ehrlich K.C., Scharfenstein L.L., Cotty P.J. (2012). Identification of genetic defects in the atoxigenic biocontrol strain Aspergillus flavus K49 reveals the presence of a competitive recombinant group in field populations. Int. J. Food Microbiol..

[100] Chang P.-K., Horn B.W., Dorner J.W. (2005). Sequence breakpoints in the aflatoxin biosynthesis gene cluster and flanking regions in nonaflatoxigenic Aspergillus flavus isolates. Fungal Genet. Biol..

[101] Lee Y.-H., Tominaga M., Hayashi R., Sakamoto K., Yamada O., Akita O. (2006). Aspergillus oryzae strains with a large deletion of the aflatoxin biosynthetic homologous gene cluster differentiated by chromosomal breakage. Appl. Microbiol. Biotechnol..

[102] Hsiang T., Baillie D.L. (2005). Comparison of the Yeast Proteome to Other Fungal Genomes to Find Core Fungal Genes. J. Mol. Evol..

[103] Cherayil B.J. (2010). The role of iron in the immune response to bacterial infection. Immunol. Res..

[104] Beach R.H., Sulser T.B., Crimmins A., Cenacchi N., Cole J., Fukagawa N.K., Mason-D’Croz D., Myers S., Sarofim M.C., Smith M. (2019). Combining the effects of increased atmospheric carbon dioxide on protein, iron, and zinc availability and projected climate change on global diets: A modelling study. Lancet Planet. Health.

[105] Greenshields D.L., Liu G., Wei Y. (2007). Roles of Iron in Plant Defence and Fungal Virulence. Plant Signal. Behav..

[106] Ramanan N., Wang Y.A. (2000). High-Affinity Iron Permease Essential for Candida albicans Virulence. Science.

[107] Schrettl M., Winkelmann G., Haas H. (2004). Ferrichrome in Schizosaccharomyces pombe—An iron transport and iron storage compound. BioMetals.

